# Prevalence of Hypertension and Associated Factors in Lubumbashi City, Democratic Republic of Congo: A Community-Based Cross-Sectional Study

**DOI:** 10.1155/2021/6674336

**Published:** 2021-04-07

**Authors:** Jacques Mbaz Musung, Placide Kambola Kakoma, Clarence Kaut Mukeng, Stéphane Lubamba Tshimanga, Jeef Paul Munkemena Banze, Nathalie Kayomb Kaj, Martin Kazadi Kamuna, Jimmy Kasali Mwamba, Dophra Ngoy Nkulu, Philippe Bianga Katchunga, Olivier Mukuku, Emmanuel Kiyana Muyumba

**Affiliations:** ^1^Department of Internal Medicine, University of Lubumbashi, Lubumbashi, Congo; ^2^Department of Public Health, University of Lubumbashi, Lubumbashi, Congo; ^3^Provincial Division of Health, Haut-Katanga Province, Lubumbashi, Congo; ^4^Sendwe Hospital, Lubumbashi, Congo; ^5^Department of Public Health, University of Kolwezi, Kolwezi, Congo; ^6^Faculty of Medicine, Catholic University of Bukavu, Bukavu, South-Kivu, Congo; ^7^Department of Research, Institu Supérieur des Techniques Médicales de Lubumbashi, Lubumbashi, Congo

## Abstract

**Background:**

Hypertension is the leading cause of cardiovascular disease morbidity and mortality worldwide. Its struggle involves knowing its prevalence. Insufficient data on hypertension in adults in Lubumbashi, Democratic Republic of Congo (DRC), prompted the conduct of this study. The objectives were to determine the prevalence of hypertension and to identify the associated factors in adults in Lubumbashi. *Methodology*. A cross-sectional study was carried out among 6,708 adults from October 15^th^ to November 24^th^, 2018, in Lubumbashi. Anthropometric data, lifestyle, and medical history were collected. Hypertension was defined when the mean of the last two blood pressure (BP) measurements was greater than or equal for systolic (SBP) at 140 mmHg and for diastolic (DBP) at 90 mmHg or a history of taking antihypertensive medication whatever the value of the BP. Logistic regression was used to identify the relative effects of hypertension risk factors and all statistical tests were declared significant at a *p* value <0.05.

**Results:**

The female participants numbered 4479 (66.8%). The mean age of all participants was 47.9 ± 16.5 years. The mean SBP and DBP were 128.4 ± 25.9 mmHg and 79.1 ± 15.3 mmHg, respectively. The overall prevalence of hypertension was 33.6%. This prevalence was statistically higher in women than in men (34.5% vs. 31.7%; *p*=0.024). After logistic regression, the risk of hypertension increased with age >50 years (aOR = 5.85 [5.19–6.60]), overweight (aOR = 1.25 [1.11–1.41]), obesity (aOR = 1.25 [1.11–1.41]), central obesity (aOR = 1.37 [1.16–1.61]), diabetes mellitus (aOR = 2.19 [1.63–2.95]), alcohol consumption (aOR = 1.21 [1.05–1.39]), nonconsumption of vegetables (aOR = 1.35 [1.02–1.80]), and history of stroke (aOR = 2.57 [1.88–3.51]). Hypertension was inversely associated with being underweight (aOR = 0.68 [0.53–0.87]).

**Conclusion:**

The prevalence of hypertension in the city of Lubumbashi is high as in other cities of the DRC and Africa. This situation requires the implementation of prevention, detection, and treatment programs for hypertension.

## 1. Introduction

Hypertension is the biggest cause of morbidity and mortality worldwide [1] and the biggest contributor to cardiovascular disease [2]. This is linked to the direct consequences of hypertension on target organs: coronary artery disease, heart failure, arterial disease, renal failure, stroke, and dementia [3, 4]. It affects over a billion people worldwide [3] (24% men and 20% women [5]) and is responsible for around 9.4 million deaths each year [6]. Its incidence is increasing in developing countries. The highest prevalence of hypertension across WHO regions is found in Africa with 30% of all adults combined [6, 7]. In the Democratic Republic of Congo (DRC), according to several studies [8–15], the prevalence of hypertension varies between 9.9 and 49.3%.

Environmental factors such as overweight/obesity, excessive alcohol consumption, dietary salt intake, stress, and insufficient physical activity play an important role in the increase in the prevalence of hypertension [16].

A global survey, called Measurements for the Month of May 2017 [17], on BP in participating adults, was conducted in May 2017 by the International Society of Hypertension (ISH) and the World Hypertension League (WHL) to screen for hypertension. This opportunity was an opportunity to update the epidemiological data of hypertension in the city of Lubumbashi (DRC).

The objective of the study was to estimate the prevalence and factors related to hypertension among adults who are 18 years and above in Lubumbashi city in DRC.

## 2. Material and Methods

Based on the worldwide survey, called Measurements of the Month of May 2017 (MMM17), on BP in adult subjects [17], conducted by the International Society of Hypertension (ISH) and the World Hypertension League (WHL), the sample of one percent of the population of adults aged 18 and over was selected for the study. In 2017, according to statistics from the Provincial Health Division of Haut-Katanga Province, the city of Lubumbashi had 2,742,921 inhabitants through its 11 health zones (HZ) including 1,215,114 adults. Thus, the study sample was set at 12,151 adults distributed proportionately in proportion to each HZ. From October 15^th^ to November 24^th^, 2018, a study was carried out in the 11 HZ of Lubumbashi with 7,142 volunteers out of the 12,151 expected subjects, that is, 58.8% participation rate. Four hundred and thirty-four participants were not included, including 347 women who had reported being pregnant, 23 subjects under the age of 18, and 64 subjects who had incomplete or aberrant data. Thus, the analysis involved 6,708 volunteers, of which 4,479 (66.8%) were female. Free, informed, and written consent was obtained from volunteer subjects before their participation in the survey. Approval for the conduct of the study was obtained from the Medical Ethics Committee of the University of Lubumbashi (UNILU/CEM/091/2018 of June 14, 2018), the Chief Doctor of the Haut-Katanga Provincial Health Division (Reference term of June 19, 2018), and the respective Chief Doctors of HZ.

Data were collected by a team of two trained and supervised health workers, within fixed health centers and selected according to their infrastructures, by combining the administration of a questionnaire and BP and anthropometric measurements. The BP measurements (expressed in mmHg), along with that of the heart rate [HR] (expressed in beats per minute [bpm]) were taken after a 5-minute rest, with a clinically validated Omron IT® automatic oscillometric blood pressure monitor. The participant was in a seated position with the back supported and the right arm resting comfortably on a table, the cuff at heart level, feet resting on the floor, and legs uncrossed.

Each participant was subjected to three BP measurements on the same day. SBP and DBP (as well as HR) measurements were averaged over the last two of three BP measurements taken 1 minute apart, and this average was considered for statistical analyzes.

Hypertension was defined as mean measured BP (mean of last 2 of 3 readings) ≥140 mmHg systolic and/or 90 mmHg diastolic, or self-reported use of medications for hypertension. The measurement of anthropometric variables (weight, height, and waist circumference), with a calculation of the body mass index (BMI) was carried out during the survey. Overweight and obesity were defined by a BMI ≥25 kg/m2 and ≥30 kg/m2, respectively [18]. Waist circumference ≥88 cm and ≥102 cm defined abdominal obesity in women and men, respectively [18].

Pregnant women were not included in the study.

The data collected had been encoded in duplicate, in order to avoid data entry errors, in Epi Info version 7.2. All analyzes were performed using Stata 12 software. Data for quantitative variables were presented as the mean with its standard deviation as appropriate and for qualitative variables as counts and percentage. Pearson's chi-square test was used to compare proportions and Student's *t*-test for comparison of means between two sexes. To estimate the probability of hypertension based on factors related, bivariate analysis was performed followed by multiple logistic regression. *p* value <0.05 was considered statistically significant.

## 3. Results

### 3.1. Background Characteristics of the Study Subjects

Table 1 summarizes the background characteristics of the study subjects. The mean age of all participants was 47.9 ± 16.5 years. The mean SBP and DBP were 128.4 ± 25.9 mmHg and 79.1 ± 15.3 mmHg, respectively, while the mean HR was 76.0 ± 12.6 bpm. Regarding the mean age of participants, no statistically significant difference was observed between female and male subjects (48.0 ± 16.6 vs. 47.8 ± 16.4 years; *p* = 0.547). Height (168.5 ± 8.2 cm vs. 159.3 ± 7.8 cm; *p* < 0.0001) and weight (65.7 ± 12.4 kg vs. 64.8 ± 15.2 kg; *p* < 0.0001) were significantly higher in males than females. On the other hand, the waist circumference (87.4 ± 13.6 cm vs. 83.0 ± 10.7 cm; *p* < 0.0001) and the BMI (25.5 ± 5.9 kg/m^2^ vs. 23, 1 ± 4.0 kg/m^2^; *p* < 0.0001) were significantly higher in women than in men.

The mean HR (77.6 ± 12.4 bpm vs. 72.8 ± 12.5 bpm; *p* < 0.0001) and DBP (79.3 ± 15.4 mmHg vs. 78.6 ± 15.0 mmHg; *p*=0.047) were significantly higher in female participants than in male participants. In contrast, men had a statistically more significant mean SBP (129.4 ± 23.4 mmHg vs. 127.8 ± 27.1 mmHg; *p*=0.013).

Personal history of stroke (3.3% vs. 3.1%; *p* < 0.0001) and regular vegetable consumption (96.8% vs. 95.2%; *p*=0.018) were statistically more significant in women than in men. On the other hand, compared to women, men had high proportions of alcohol consumption (37.5% vs. 18.8%; *p* < 0.0001), smoking (13.4% vs. 1.3%; *p* < 0.0001), and physical activity (38.9% vs. 15.5%; *p* < 0.0001).

Fruit consumption, personal history of diabetes mellitus, and personal history of stroke were not statistically significant between the sexes (*p* > 0.05).

### 3.2. Blood Pressure according to Age and Sex

**Figure 1:**
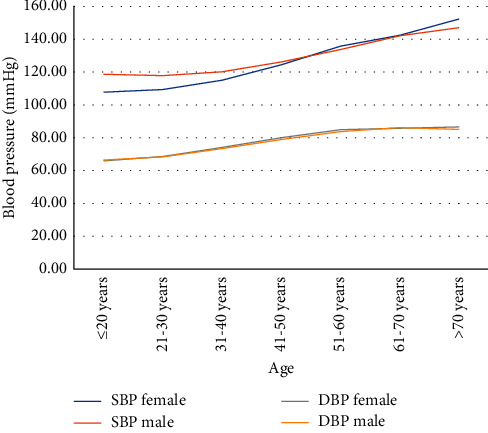
Trend of systolic and diastolic blood pressure by age and sex.

SBP and DBP increase with age in both sexes (Figure 1). Based on the mixed linear model, the overall association between age and SBP in men and women showed a linear increase, with the means of SBP in women exceeding the means of SBP in men of 50 years of age. For DBP, the relationship shows an inverted U-shape, with the highest levels at 51–60 years and with lower BP in women than in men up to age 70.

### 3.3. Prevalence of Hypertension

Table 2 shows that the overall prevalence of hypertension was 33.6% (2251/6708). Excluding participants who were taking an antihypertensive drug (9.3%), this prevalence was 26.7% (1625/6082). The distribution of this overall prevalence of hypertension shows that the proportion of hypertensive women (34.5%; 1544/4479) was higher (*p*=0.024) than that of men (31.7%; 707/2229). When distributing the prevalence of hypertension according to the HZs surveyed, it was higher in the Kamalondo HZ (50.4%) and lower in the Tshamilemba HZ (23.2%).

### 3.4. Overall Prevalence of Hypertension by Age Group and Sex

**Figure 2:**
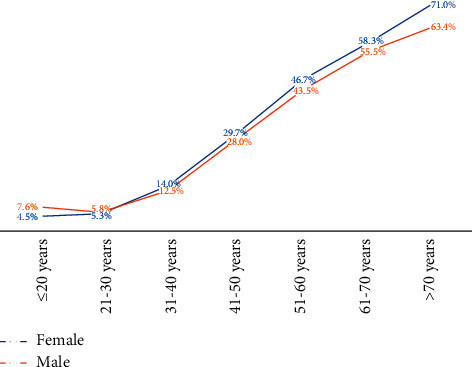
Overall prevalence of hypertension by age group and sex.

The distribution of the overall prevalence of hypertension increases with age for both sexes, ranging from 5.4% in subjects aged ≤20 years to 68.4% in subjects aged ˃70 years (*p* < 0.001). Females subjects aged ≤30 years had a lower prevalence of hypertension than males, while after age 30, the prevalence was higher in women (Figure 2).

### 3.5. Factors Associated with Hypertension

Table 3 describes the proportion of hypertensive subjects among carriers of other factors associated with hypertension. For both sexes, the bivariate analysis indicated that the risk of hypertension increased with age >50 years (cOR = 6.21 [5.55–6.96]; *p* < 0.000), in the presence of overweight (cOR = 1.71 [1.51–1.92]; *p* < 0.0001), obesity (cOR = 2.31 [1.99–2.67]; *p* < 0.0001), abdominal obesity (cOR = 2.13 [1.91–2.36]; *p* < 0.0001), personal history of diabetes mellitus (cOR = 3.44 [2.63–4.51]; *p* < 0.0001), family history of hypertension (cOR = 0.63 [0.56–0.71]; *p* < 0.0001), personal history of stroke (cOR = 3.67 [2.76–4.86]; *p* < 0.0001) and physical inactivity (cOR = 1.25 [1.11–1.41]; *p* < 0.0001).

In multivariate analysis (Table 3), the risk of hypertension increased with age >50 years (aOR = 5.85 [5.19–6.60]; *p* < 0.0001), overweight (aOR = 1.25 [1.11–1.41]; *p* < 0.0001), obesity (aOR = 1.25 [1.11–1.41]; *p* < 0.0001), abdominal obesity (aOR = 1.37 [1.16–1.61]; *p* < 0.0001), personal history of diabetes mellitus (aOR = 2.19 [1.63–2.95]; *p* < 0.0001), consumption of alcohol (aOR = 1.21 [1.05–1.39]; *p*=0.007), nonconsumption of vegetables (aOR = 1.35 [1.02–1.80]; *p*=0.038), and personal history of stroke (aOR = 2.57 [1.88–3.51]; *p* < 0.0001). Factors not included in the model were gender (*p*=0.110), physical inactivity (*p* < 0.461), family history of hypertension (*p* < 0.061), cigarette smoking (*p*=0.751), and fruit consumption (*p*=0.957) whose influence was not significant.

## 4. Discussion

The overall prevalence of hypertension in the current survey was 33.6%. In comparison with the prevalences found in previous studies carried out in urban areas among Congolese adults in the DRC, this prevalence was higher than 28.3% and 30.9% reported, respectively, by Atoba et al. in Kisangani [11] and Bayauli et al. in Kinshasa [12]; less than 41.4%, 41.9%, and 49.3%, respectively, found by Kachunga et al. in Bukavu [13], Kusuayi-Mabele et al. in Kinshasa [14], and Kabamba et al. in Lubumbashi [15]. Compared to authors from Sub-Saharan Africa, the prevalence of hypertension reported in this study was similar to 34.5% reported by Savarino et al. in Angola [19]. However, it is higher than those observed in the surveys by Kramoh et al. in Côte d'Ivoire (20.4%) [20], Henry et al. in Malawi (22.3%) [21], Elijah et al. in Kenya (24.6%) [22], Fastone et al. in Zambia (25.9%) [23], and Anastase et al. in Cameroon (29.2%) [24]. On the other hand, it is lower than that noted in the survey by Bertrand et al. in the Republic of Congo (41%) [25]. The prevalence of hypertension in the present survey was within the range of the prevalence observed in the global surveys of MMM17 and MMM18, respectively, 34.9% and 33.4% [26,27]. Several reasons may justify these differences in prevalence between these studies: different methodologies (type of blood pressure monitor used to measure BP, number of BP measurements, target populations, and sample size), differences in climatic conditions, ethnicity, demographic, socioeconomic, and so forth.

As in other studies [10–13,26–30], the present study had shown that advanced age was independently associated with hypertension and the probability of developing hypertension increased with age (aOR = 5.85; *p*=0.000). This may be explained by the fact that, with increasing age, the walls of the larger arteries stiffen mainly due to arteriosclerotic structural changes, calcification, and increased peripheral vascular resistance of the small arteries [31].

A highly significant association (*p* < 0.0001) was observed between overweight/obesity, central obesity, and hypertension. Several previous studies carried out in the DRC [10–14], in Sub-Saharan Africa, and in the world [19–27] had demonstrated this significant association between overweight/obesity and abdominal obesity with the increase in hypertension. The more frequent overweight and obesity in the urban population reflects an epidemiological transition linked to dietary changes and to lifestyle modifications with a strong component linked to physical inactivity and stress [13, 29]. Urbanization in developing countries has been associated with lifestyle changes that lead to increased consumption of high-calorie foods and has led to several environmental factors which generate a less active rhythm of life which is summed up in an increase in sedentary behavior during professional activities, a reduced practice of physical activity during hobbies time, and an increase in the use of means passive transport [32]. Some authors indicate that adverse health consequences are associated with increased adiposity rather than increased body weight [33, 34]; evidence has shown that a higher BMI represents 75% of the risk of primary hypertension which is mediated by increased renal tubular reabsorption of sodium which impairs natriuresis [35]. As in several community surveys conducted in different countries [13, 21, 23, 26, 27, 29, 30], this survey found that a BMI ≥ 25 kg/m^2^ was associated with hypertension. We also found that abdominal obesity was linked to hypertension independent of body mass index or generalized obesity. Consistent with this study, other studies also found that hypertension was significantly associated with abdominal obesity [13, 14, 30, 36, 37]. However, an Ethiopian study of adults in Jigjiga City [38] reported that abdominal obesity was not associated with hypertension.

Participants with diabetes had a 2.19-fold higher risk of being hypertensive (aOR = 2.19 [1.63–2.95]; *p* < 0.0001). This finding is similar to previous studies [13, 19–27]. The pathogenesis of hypertension in diabetics is complex and involves strong interactions between genetic predisposition and various environmental and biological factors, such as poor diet, sedentary lifestyle, calcium retention, abdominal obesity, vegetative imbalances, premature arterial hardening, and endothelial dysfunction [39]. Depending on whether it is type 1 or type 2 diabetes, the pathophysiology is different. In type 1 diabetes, high blood pressure is often the result of an underlying kidney disease; in type 2 diabetes, it is more often essential and occurs in a context of multiple metabolism and insulin resistance. In all cases, hypertension worsens the prognosis of diabetic subjects by increasing the cardiovascular risk and accelerating the onset of degenerative complications. By its frequency in the diabetic population, hypertension, therefore, plays a major role in the onset of chronic complications of diabetes mellitus [39]. Alcohol consumption was independently associated with hypertension (aOR = 1.21 [1.05–1.39]; *p*=0.007) in the present study as well as that of Atoba et al. [11]. On the other hand, Bayauli et al. [12] and Katchunga et al. [13] did not demonstrate this association. The main factor responsible for hypertension in patients who drink alcohol heavily is the activation of the sympathetic nervous system, which completely suppresses the vasodilator effect of alcohol. This activation is due to the increased production of hypothalamic corticoliberin (corticotropin-releasing factor). This hypothesis is confirmed by the suppression of hypertension induced by alcohol during the simultaneous administration of dexamethasone which exerts a feedback inhibition on the secretion of corticotropin-releasing factor by the hypothalamus [40, 41]. This results in hypersecretion of catecholamines (in the central nervous system as well as the kidneys and adrenals), cortisol, plasma angiotensin, and aldosterone by increasing plasma renin activity [42]. In addition, alcohol is said to have a direct effect on smooth muscle cells. In fact, there is habituation to the acute vasodilator effect of alcohol with an exaggerated sensitization to the vasoconstrictor effects of vasomotor amines, thus leading to an increase in peripheral vascular resistance and arterial hypertension [43]. Finally, on an experimental level, the ionic movements in the smooth muscle cells are modified under the effect of alcohol with, in particular, an increase in calcium entry into the muscle cells and a decrease in the quantity of magnesium in these cells, calcium having an extremely important constricting role [42, 43].

Contrary to certain authors [13, 29], the subjects who declared that they did not regularly consume vegetables had developed significantly more hypertension than those who consumed them (aOR = 1.35 [1.02–1.80]; *p*=0.038). A recent Ethiopian study found that increasing the consumption of fruits and vegetables reduced the likelihood of hypertension among study participants [44]. Likewise, meta-analysis reports have shown that increasing fruit and vegetable consumption prevents the risk of developing hypertension [45, 46]. However, the questionnaire of our study did not allow us to quantify this consumption of vegetables and fruits in relation to the recommendations which recommend a daily consumption of 4 to 5 fruits and vegetables per day [47–49] and finally to draw a conclusion.

Interpretation of the results of this study should consider certain limitations. First, because the study is cross-sectional, it prevents the establishment of any relationship between the results and the associated factors. Second, the single measure of BP might underestimate or overestimate their true values and the prevalence of hypertension. Third, some factors associated with hypertension in this study were based on the respondents' statements and not on their measurements. Another limitation of this study was the inability to assess some factors, for example, educational level and history of kidney disease, history of hypercholesterolemia, hyperuricemia, and type of medication such as nonsteroidal anti-inflammatory drugs and steroids, which have reportedly been associated with hypertension. The future study must include these variables.

## 5. Conclusion

The prevalence of hypertension in the city of Lubumbashi is high as in other cities of the DRC and Africa. The high prevalence of hypertension was associated with increased age, overweight, obesity, abdominal obesity, diabetes mellitus, alcohol consumption, and personal history of stroke. These results highlight the need for prevention, detection, and management programs for hypertension and associated factors. Community intervention directed towards changing or modifying behavioral risk factors should be developed to address individual level risk factors. Interventions that target modifiable risk factors of hypertension might decrease blood pressure, and even preventing the development of hypertension should be implemented. Evidence-based prevention and management recommendations and guidelines including lifestyle modifications need to be adopted in Lubumbashi in DRC.

## Figures and Tables

**Table 1:** The background characteristics of the study subjects (*n* = 6,708).

Variable	Female *n* = 4,479 (66.8%)	Male *n* = 2,229 (33.2%)	Total *N* = 6,708	*p* value
Age (years), mean ± SD	48.0 ± 16.6	47.8 ± 16.4	47.9 ± 16.5	0.547
Weight (kg), mean ± SD	64.8 ± 15.2	65.7 ± 12.4	65.1 ± 14.3	0.006
Height (cm), mean ± SD	159.3 ± 7.8	168.5 ± 8.2	162.3 ± 9.0	<0.0001
Waist circumference (cm), mean ± SD	87.4 ± 13.6	83.0 ± 10.7	85.9 ± 12.9	<0.0001
BMI (kg/m^2^), mean ± SD	25.5 ± 5.9	23.1 ± 4.0	24.7 ± 5.4	<0.0001
SBP (mmHg), mean ± SD	127.8 ± 27.1	129.4 ± 23.4	128.4 ± 25.9	0.013
DBP (mmHg), mean ± SD	79.3 ± 15.4	78.6 ± 15.0	79.1 ± 15.3	0.044
HR (bpm), mean ± SD	77.6 ± 12.4	72.8 ± 12.5	76.0 ± 12.6	<0.0001
Alcohol consumption, n (%)	840 (18.8)	835 (37.5)	1675 (25.0)	<0.0001
Cigarette smoking, n (%)	59 (1.3)	299 (13.4)	358 (5.4)	<0.0001
Vegetables consumption, n (%)	4321 (96.8)	1446 (95.2)	6444 (96.1)	0.018
Fruit consumption, n (%)	2871(64.1)	1397 (62.7)	4268 (63.6)	0.264
Physical activity, n (%)	692 (15.5)	868 (38.9)	1560 (23.3)	<0.0001
History of diabetes mellitus, n (%)	155 (3.5)	79 (3.5)	234 (3.5)	0.916
History of stroke, n (%)	146 (3.3)	70 (3.1)	216 (3.2)	0.851
Family history of hypertension, n (%)	1506 (33.6)	641 (28.9)	2147 (32.0)	<0.0001

SD: standard deviation; BMI: body mass index; SBP: systolic blood pressure; DBP: diastolic blood pressure; HR: heart rate; bpm: beats per minute.

**Table 2:** Distribution of the overall prevalence of hypertension according to the health zones of Lubumbashi.

Health zone	Participants/expected *n* (%)	Participants with hypertension *n* (%)	Participants taking antihypertensive drugs *n* (%)
Katuba	469/659 (71.2)	117/469 (37.7)	60/469 (12.7)
Kampemba	1216/2240 (54.3)	478/1216 (39.3)	123/1216 (10.1)
Kamalondo	133/146 (91.1)	67/133 (50.4)	38/133 (28.5)
Kenya	655/888 (73.8)	267/655 (40.8)	64/655 (9.7)
Kisanga	987/1577 (62.6)	307/987 (31.1)	71/987 (7.2)
Kowe	194/251 (72.3)	65/194 (33.5)	13/194 (6.7)
Lubumbashi	651/1100 (59.2)	196/651 (30.1)	69/651 (10.5)
Mumbunda	565/1332 (42.4)	186/565 (32.9)	43/565 (7.6)
Ruashi	1024/2218 (46.2)	280/1024 (27.3)	69/1024 (6.7)
Tshamilemba	475/1085 (43.8)	110/475 (23.2)	41/475 (8.6)
Vangu	339/655 (51.7)	118/339 (34.8)	35/339 (10.3)
Total	6708/12151 (55.2)	2251/6708 (33.6)	626/6708 (9.3)

**Table 3:** Multiple logistic regression analysis of determinants of hypertension among study participants.

Variable	Total (*N* = 6,708)	Hypertensive participants (*n* = 2,251)		No hypertensive participants (*n* = 4,457)		Crude OR [IC95%]	*p* value	Adjusted OR [IC95%]	*p* value
*Age*									
≤50 years	3689	600	(16.26%)	3089	(83.74%)	1.00	0.000	1.00	0.000
>50 years	3019	1651	(54.69%)	1368	(45.31%)	6.21 [5.55–6.96]		5.85 [5.19–6.60]	

*Sex*									
Female	4479	1544	(34.47%)	2935	(65.53%)	1.13 [1.02–1.26]	0.024	0.89 [0.77–1.02]	0.110
Male	2229	707	(31.72%)	1522	(68.28%)	1.00		1.00	

*BMI*									
Underweight	439	103	(23.46%)	336	(76.54%)	0.79 [0.62–0.99]	0.047	0.68 [0.53–0.87]	0.002
Normal	3550	992	(27.94%)	2558	(72.06%)	1.00		1.00	
Overweight	1731	689	(39.80%)	1042	(60.20%)	1.71 [1.51–1.92]	0.000	1.44 [1.24–1.67]	0.000
Obesity	988	467	(47.27%)	521	(52.73%)	2.31 [1.99–2.67]	0.000	1.98 [1.62–2.44]	0.000

*Abdominal obesity*									
No	4524	1264	(27.94%)	3260	(72.06%)	1.00	0.000	1.00	0.000
Yes	2184	987	(45.19%)	1197	(54.81%)	2.13 [1.91–2.36]		1.37 [1.16–1.61]	

*History of diabetes mellitus*									
Absent	6474	2105	(32.51%)	4369	(67.49%)	1.00	0.000	1.00	0.000
Present	234	146	(62.39%)	88	(37.61%)	3.44 [2.63–4.51]		2.19 [1.63–2.95]	

*Familial history of hypertension*									
Absent	4561	1675	(36.72%)	2886	(63.28%)	1.00	0.000	1.00	0.061
Present	2147	576	(26.83%)	1571	(73.17%)	0.63 [0.56–0.71]		0.88 [0.78–1.00]	

*History of stroke*									
Absent	6492	2113	(32.55%)	4379	(67.45%)	1.00	0.000	1.00	0.000
Present	216	138	(63.89%)	78	(36.11%)	3.67 [2.76–4.86]		2.57 [1.88–3.51]	

*Cigarette smoking*									
No	6350	2136	(33.64%)	4214	(66.36%)	1.00	0.555	1.00	0.751
Yes	358	115	(32.12%)	243	(67.88%)	0.93 [0.74–1.17]		0.98 [0.75–1.28]	

*Alcohol consumption*									
No	5033	1679	(33.36%)	3354	(66.64%)	1.00	0.553	1.00	0.007
Yes	1675	572	(34.15%)	1103	(65.85%)	1.03 [0.92–1.16]		1.21 [1.05–1.39]	

*Physical activity*									
Yes	1560	465	(29.81%)	1095	(70.19%)	1.00	0.000	1.00	0.461
No	5148	1786	(34.69%)	3362	(65.31%)	1.25 [1.11–1.41]		1.06 [0.91–1.22]	

*Vegetable consumption*									
Yes	6444	2148	(33.33%)	4296	(66.67%)	1.00	0.055	1.00	0.038
No	264	103	(39.02%)	161	(60.98%)	1.28 [0.99–1.65]		1.35 [1.02–1.80]	

*Fruit consumption*									
Yes	4268	1409	(33.01%)	2859	(66.99%)	1.00	0.212	1.00	0.957
No	2440	842	(34.51%)	1598	(65.49%)	1.07 [0.96–1.19]		0.99 [0.88–1.12]	

SD: standard deviation; BMI: body mass index; SBP: systolic blood pressure; DBP: diastolic blood pressure; HR: heart rate; bpm: beats per minute.

## Data Availability

The datasets used and analyzed during the current study are available from the corresponding author (OM) on reasonable request.

## Abbreviations

aOR: Adjusted odds ratio
BP: Blood pressure
bpm: Beats per minute
cOR: Crude odds ratio
DBP: Diastolic blood pressure
DRC: Democratic Republic of Congo
HR: Heart rate
SBP: Systolic blood pressure
HZ: Health zone.
